# Gremlin-2 inhibits hepatocellular carcinoma progression by suppressing Akt/MEK–RPL23 signaling pathway

**DOI:** 10.1016/j.gendis.2026.102074

**Published:** 2026-02-09

**Authors:** Dayeon Lim, Jayeon Park, World Gil, Jiwoo Jung, Minji Kwon, Seoha Jo, Ye Hwang Cheong, Mee-Hyun Lee, Hee Sun Park, Sin-Aye Park

**Affiliations:** a Department of Medical Sciences, Graduate School, Soonchunhyang University, Asan 31538, Republic of Korea; b Department of Biomedical Laboratory Science, College of Medical Sciences, Soonchunhyang University, Asan 31538, Republic of Korea; c Institute for Molecular Metabolism Innovation, Soonchunhyang University, Asan 31538, Republic of Korea; d Drug Discovery Research Laboratories, Dong-A ST Co., Ltd., Yongin 17073, Republic of Korea; e College of Korean Medicine, Dongshin University, Naju 58245, Republic of Korea; f Division of Pulmonology, Department of Internal Medicine, College of Medicine, Chungnam National University, Daejeon 35015, Republic of Korea

Hepatocellular carcinoma (HCC) is a heterogeneous malignancy that arises from multiple risk factors, including chronic hepatitis B or C infection, excessive alcohol consumption, chronic liver disease, and obesity. Despite significant advances in understanding its molecular pathogenesis, the regulatory mechanisms driving HCC progression remain incompletely defined. Gremlin-2 (GREM2), a member of the DAN family and a functional antagonist of bone morphogenetic proteins, regulates both metabolic and tumor-related pathways.^1^, ^2^, ^3^ It suppresses adipogenesis through activation of the Wnt/β-catenin signaling pathway and, as shown in our previous work, inhibits breast cancer cell proliferation by reducing adipocyte-derived adipokine expression and secretion.^3^ Moreover, empagliflozin has been reported to alleviate fibrosis in nonalcoholic fatty liver disease by down-regulating miRNA-34a-5p, a negative regulator of GREM2.^4^ Given the strong association between metabolic dysregulation and liver cancer development, GREM2 is likely to play an important regulatory role in HCC, although its function remains largely unexplored.

To examine the role of GREM2 in HCC, we first assessed its expression in HCC tissues and cell lines. Analysis of public datasets (GEPIA and TNMplot) revealed markedly reduced GREM2 mRNA levels in HCC and metastatic liver tissues compared with normal liver tissues (Fig. 1A and B). Consistently, immunofluorescent staining of tissue microarrays demonstrated weaker GREM2 signals in tumor sections relative to non-tumor controls (Fig. S1A). Expression atlas data further indicated that GREM2 transcript levels were consistently lower across all 24 HCC cell lines examined compared with the proliferation marker MKI67 (Fig. S1B). Reduced GREM2 expression was associated with poorer patient outcomes, with overall survival hazard ratio (HR) 0.65 (*P* = 0.024, *n* = 322) and disease-specific survival HR 0.62 (*P* = 0.048, *n* = 315) (Fig. 1C). To evaluate functional consequences, we generated stable GREM2-overexpressing HepG2 and Huh7 cell lines (Fig. S1C and S1D). This overexpression significantly reduced cell viability (Fig. 1D), colony formation (Fig. S1E and S1F), and migration capabilities (Fig. S1G and S1H) of HCC cells. Cell-cycle analysis revealed G2/M phase accumulation with a corresponding decrease in G0/G1 population (Fig. 1E; Fig. S1I), along with down-regulation of key cell-cycle regulators (Fig. S1J). Suppression of GREM2 expression increased HCC cell viability; however, this effect was less pronounced compared with overexpression experiments, likely due to the inherently low basal levels of GREM2 in these cells (Fig. S1K). Notably, GREM2 did not influence lipogenesis in HCC cells (Fig. S1L and S1M). These results indicate that GREM2 is markedly down-regulated in HCC and functions as a suppressor of tumor cell proliferation, acting independently of its role in regulating adipogenesis.

**Figure 1:**
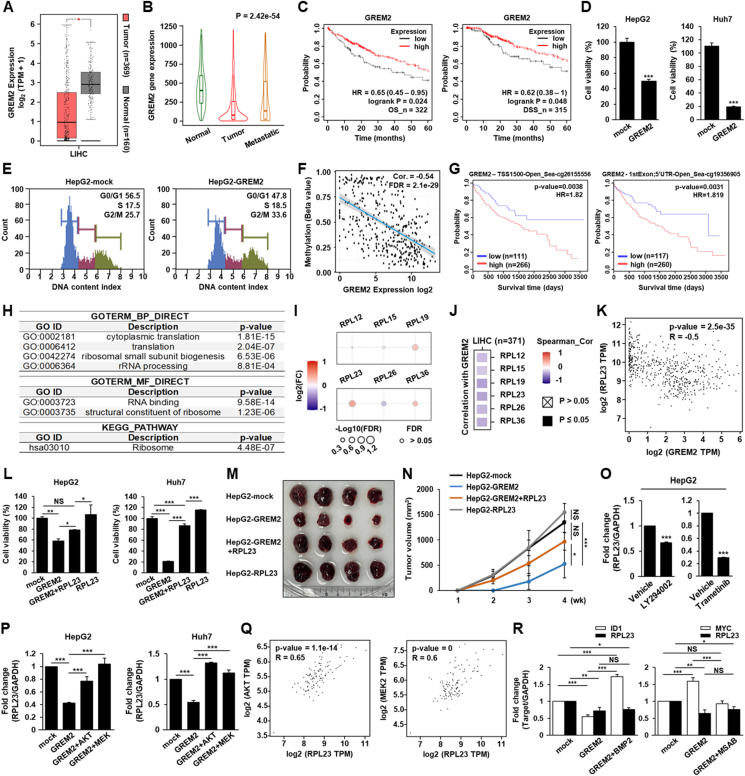
Promoter hypermethylation-mediated inhibition of GREM2 mRNA expression contributes to poor survival in hepatocellular carcinoma (HCC) patients, whereas overexpression inhibits cell proliferation by blocking the Akt/MEK–RPL23 signaling pathway. **(A)** GREM2 transcript levels were compared between TCGA-LIHC tumors and combined TCGA/GTEx normal liver tissues using GEPIA2. **(B)** TNMplot gene-chip data were used to evaluate GREM2 expression in normal liver (*n* = 379), primary HCC (*n* = 806), and metastatic lesions (*n* = 24). **(C)** Kaplan–Meier survival curves show overall survival and disease-specific survival according to GREM2 expression. **(D)** Cell viability after 48 h of culture in 96-well plates was measured by MTT assay. **(E)** Cell-cycle distribution was analyzed with a Muse™ Cell Cycle Kit. **(F)** Spearman correlation between GREM2 expression and promoter methylation in TCGA-LIHC was determined. **(G)** MethSurv Kaplan–Meier plots show HCC patient survival according to methylation of individual GREM2 CpG sites. **(H)** Ribosome-related pathways enriched among negatively correlated genes are summarized. **(I)** The expression of RP genes was compared between TCGA-LIHC tumors and normal tissues using GSCA. **(J)** Spearman correlations between GREM2 and RP genes in TCGA-LIHC were analyzed using TIMER2.0. **(K)** Correlation between GREM2 and RPL23 expression was examined using GEPIA2 with TCGA-LIHC, TCGA-normal, and GTEx-liver datasets. **(L)** MTT assay assessed cell viability after 48 h in cell lines overexpressing GREM2, RPL23, or both. **(M, N)** Representative xenograft tumors and mean tumor volumes from mice injected with the indicated cell lines are shown. **(O)** Quantitative PCR quantified target genes after 24 h treatment with LY294002 (25 μM) or trametinib (1 μM). **(P)** RPL23 levels were measured by quantitative PCR in each cell line. **(Q)** Spearman correlations between RPL23 and AKT or MEK2 mRNA in GTEx-liver were determined using GEPIA2. **(R)** RPL23 expression was assessed by quantitative PCR after BMP2 overexpression or MSAB (10 μM) treatment in GREM2-overexpressing HepG2 cells.

Because promoter CpG methylation is a common mechanism for transcriptional repression, we examined whether GREM2 down-regulation was associated with hypermethylation. GSCA database analysis revealed the strongest inverse correlation between GREM2 mRNA expression and promoter methylation in HCC (TCGA-LIHC) compared with other cancer types (Fig. S2A). In TCGA-LIHC samples, GREM2 transcript levels were negatively correlated with both promoter methylation (Fig. 1F) and DNA methyltransferase expression (Fig. S2B). UCSC Genome Browser analysis revealed that DNMT3A expression showed the strongest positive correlation with methylation levels of three CpG sites in the GREM2 promoter (Fig. S2C and S2D). MethSurv analysis further revealed that hypermethylation of four specific GREM2 promoter CpG sites (cg26155556, cg19356905, cg11504078, and cg23210268) was significantly associated with poorer patient survival (Fig. 1G; Fig. S2E). Treatment of HCC cells with the demethylating agent decitabine induced dose-dependent increases in GREM2 mRNA expression (Fig. S2F) and a corresponding decrease in cell viability (Fig. S2G). These findings suggest that promoter hypermethylation is a key mechanism suppressing GREM2 transcription in HCC.

To identify pathways influenced by GREM2, we analyzed TCGA-LIHC RNA-sequencing data and identified 549 genes positively correlated and 1529 genes negatively correlated with GREM2 expression (|*r*| > 0.25). Functional enrichment using DAVID categorized these genes into biological processes, molecular functions, cellular components, and KEGG pathways (Fig. S3A–S3E). Many negatively correlated pathways were concentrated in translation- and ribosome-related processes (Fig. 1H), suggesting that GREM2 may regulate gene expression by modulating translation. Given the association with ribosomal pathways, we assessed whether GREM2 regulated ribosomal protein (RP) genes implicated in cancer proliferation. GSCA analysis confirmed that the expression of representative RP genes (RPL12, RPL15, RPL19, RPL23, RPL26, and RPL36) was significantly increased in HCC tissues compared with normal tissues (Fig. 1I). TIMER2.0 analysis revealed significant negative correlations between GREM2 and these RP genes in HCC tissues (Fig. 1J). In GREM2-overexpressing HCC cells, most RP genes were down-regulated, with RPL23 showing the most pronounced decrease (Fig. S4A). Across multiple datasets (TCGA-LIHC, TCGA-normal, and GTEx-liver), GREM2 and RPL23 mRNA levels were inversely correlated (Fig. 1K). GEMiCCL database analysis showed reduced GREM2 expression and elevated RPL23 expression across 11 HCC cell lines, with RPL23 levels comparable to MKI67 (Fig. S4B). Elevated RPL23 expression was correlated with poorer overall survival (HR = 1.63, *P* = 0.0065) and relapse-free survival (HR = 1.68, *P* = 0.0018) (Fig. S4C). The reductions in cell viability (Fig. 1L), colony formation (Fig. S5A), and migration (Fig. S5B and S5C) induced by GREM2 were partially rescued by RPL23 overexpression. *In vivo*, subcutaneous injection of HepG2-GREM2 cells into mice yielded significantly smaller tumors compared with HepG2-mock cells (525.76 ± 275.61 mm^3^ *vs*. 1347.34 ± 197.37 mm^3^), and co-overexpression of RPL23 restored tumor growth (968.71 ± 170.48 mm^3^) (Fig. 1M and N). Proliferation-associated gene expression, reduced by GREM2, was also restored by RPL23 (Fig. S5D). These results demonstrate that GREM2 suppresses HCC progression primarily through inhibition of RPL23.

To elucidate the mechanism underlying RPL23 repression, we examined upstream signaling pathways. Previous studies have shown that RPL23 expression can be regulated not only by mTOR but also by the MEK and PI3K/Akt signaling pathways.^5^ Treatment with Akt (LY294002) or MEK (trametinib) inhibitors significantly reduced RPL23 mRNA expression, whereas mTOR inhibition (rapamycin) produced variable effects in the two HCC cell lines (Fig. 1O; Fig. S6A and S6B). GREM2 overexpression reduced both RPL23 expression and Akt/MEK phosphorylation (Fig. S6C), and co-overexpression of Akt or MEK restored RPL23 levels (Fig. 1P). RPL23 mRNA levels were positively correlated with AKT and MEK2 in GTEx-liver tissues (Fig. 1Q). Besides, the reductions in proliferation (Fig. S6D) and migration (Fig. S6E and S6F) induced by GREM2 were partially rescued by Akt or MEK overexpression. Importantly, overexpression of BMP2 or inhibition of Wnt/β-catenin signaling by MSAB did not affect GREM2-mediated RPL23 repression, despite alterations in representative target genes (BMP2: ID1; Wnt: MYC) of each pathway (Fig. 1R). This suggests that classical signaling pathways associated with GREM2 are not involved in repressing RPL23 expression, an area that requires further investigation.

In conclusion, this study identifies GREM2 as a novel tumor suppressor that is epigenetically repressed in HCC. Promoter hypermethylation significantly down-regulated GREM2 expression in both HCC tissues and cell lines, leading to its transcriptional repression. Clinically, low GREM2 expression and promoter hypermethylation were closely associated with poor patient survival, highlighting the prognostic significance of GREM2. Functionally, GREM2 overexpression significantly suppressed HCC cell proliferation, migration, and tumor growth, demonstrating its tumor suppressive role. Transcriptome analysis revealed a previously unknown negative regulatory link between GREM2 and ribosome-related genes, particularly RPL23. Mechanistically, we demonstrated that GREM2 inhibited HCC progression by blocking the Akt/MEK-RPL23 axis, thereby interfering with oncogenic ribosome signaling. These findings provide compelling evidence that GREM2 inactivation through promoter hypermethylation contributes to HCC pathogenesis, highlighting GREM2 as a promising diagnostic biomarker and therapeutic target for future epigenetic and targeted therapeutic strategies in HCC.

## CRediT authorship contribution statement

**Dayeon Lim:** Writing – original draft, Methodology, Investigation, Data curation. **Jayeon Park:** Writing – original draft, Methodology, Investigation, Data curation. **World Gil:** Investigation, Data curation. **Jiwoo Jung:** Methodology, Data curation. **Minji Kwon:** Methodology, Data curation. **Seoha Jo:** Validation, Methodology. **Ye Hwang Cheong:** Formal analysis, Data curation. **Mee-Hyun Lee:** Project administration, Methodology. **Hee Sun Park:** Project administration, Methodology. **Sin-Aye Park:** Writing – review & editing, Supervision, Project administration, Funding acquisition, Conceptualization.

## Ethics declaration

All animal procedures were carried out in compliance with the animal care guidelines of Soonchunhyang University and were approved by the Institutional Animal Care and Use Committee (SCH25-0058).

## Funding

This work was supported by the 10.13039/501100002560Soonchunhyang University research fund and the 10.13039/501100003725National Research Foundation of Korea (NRF) grant funded by the Korean government (MIST) (No. 2022R1F1A1074226). This research was also supported by Global-Learning & Academic research institution for Master's·PhD students, and Postdocs (G-LAMP) Program of the National Research Foundation of Korea (NRF) grant funded by the Ministry of Education (No. RS-2025-25441283).

## Conflict of interests

The authors state that they have no financial or personal relationships that could have influenced the research reported in this manuscript.
