# Supplementary material for: Gremlin-2 inhibits hepatocellular carcinoma progression by suppressing Akt/MEK–RPL23 signaling pathway
Source: Genes Dis. 2026 Feb 9;13(6):102074. doi: 10.1016/j.gendis.2026.102074 (PMC13380146; doi:10.1016/j.gendis.2026.102074)
Supplement: Multimedia component 1 [file mmc1.docx]

**Materials and methods**

**Cell culture and reagents**

HCC cell lines HepG2 and Huh7 were obtained from the Korean Cell Line Bank (Republic of Korea). Cells were propagated in Dulbecco’s Modified Eagle Medium (DMEM; Corning, USA) supplemented with 10% fetal bovine serum (FBS; Thermo Fisher Scientific, USA) and 1% penicillin-streptomycin (Corning) under a humidified 5% CO₂ atmosphere at 37 °C. Primary antibodies against GREM2 (Abcam, UK), cyclin B1, cyclin D1, cyclin E2, CDK1, CDK2, CDK4, CDK6, p-AKT, AKT, and β-actin (Cell Signaling Technology, USA) were used. Anti-RPL23 antibody was from Proteintech (USA), and anti–p-MEK-1/2 and anti–MEK-1/2 antibodies were from Santa Cruz Biotechnology (USA). Chemical inhibitors included decitabine (HY-A0004) and trametinib (HY-10999R) from MedChemExpress (USA), LY294002 (S1105) and MSAB (S6901) from Selleckchem (USA), and rapamycin (553210) from Sigma-Aldrich (USA).

**Establishment of stable cell line**

HepG2 and Huh7 cells were seeded in DMEM/10% FBS until approximately 50% confluence, followed by transfection with ORF expression plasmids for GREM2 (Sino Biological, HG10283-CY, China), RPL23 (OriGene, RG210650, USA), AKT1 (Sino Biological, HG10763-CY), or MEK2 (Sino Biological, HG10678-UT) using Gen-Fection (ProGeneX BIO, Republic of Korea) according to the manufacturer’s instructions. After 48 h, transfected cells were subjected to antibiotic selection with hygromycin or neomycin (both from InvivoGen, USA) to obtain stable populations.

**Quantitative real-time PCR (qRT-PCR)**
Total RNA was isolated with TRIzol® reagent (Thermo Fisher Scientific) and reverse-transcribed using M-MLV reverse transcriptase (Enzynomics, Republic of Korea). qRT-PCR was carried out with TOPreal™ qPCR 2X Pre-Mix (Enzynomics) on a Step-One-Plus Real-Time PCR instrument (Thermo Fisher Scientific). Primer sequences are provided in Supplementary Table 1. GAPDH or ACTB served as internal controls.

**Western blotting**
Cells were lysed in lysis buffer containing protease and phosphatase inhibitors (Roche, Switzerland). Protein concentrations were determined with a BCA assay (Thermo Fisher Scientific). Proteins were separated on 8–12% SDS–polyacrylamide gels and electrotransferred to polyvinylidene difluoride membranes (Merck Millipore, USA). Membranes were incubated overnight at 4 °C with primary antibodies, followed by horseradish peroxidase–conjugated secondary antibodies (AbFrontier, Republic of Korea) for 1 h at room temperature. Signal detection was performed using Westsave Gold reagent (AbFrontier).

**Colony formation**

Cells (2 × 10⁴ in 6-well plates or 1 × 10⁴ in 12-well plates) were cultured for 7–10 days, fixed with methanol, and stained with 0.5% crystal violet. Stained colonies were solubilized in dimethyl sulfoxide, and absorbance was measured for quantification.

**Scratch-wound migration assay**
Confluent monolayers in 12-well plates were scratched using a 200 µL pipette tip to create a wound. After washing, cells were incubated with DMEM containing 10% FBS and imaged at indicated time points using an inverted phase-contrast microscope (4× objective). Wound closure was quantified with ImageJ.

**Cell-cycle profiling**
Approximately 1 × 10⁶ cells were fixed in 70% cold ethanol for 3 h at −20 °C, washed with PBS, and stained with Muse™ Cell Cycle reagent for 30 min in the dark. Cell-cycle distribution was analyzed with a Muse™ Cell Analyzer (Cytek® Biosciences, USA).

**Immunofluorescence**
Tissue sections were deparaffinized, antigen-retrieved, and permeabilized following standard protocols before incubation with primary antibodies. Alexa Fluor® 488–conjugated secondary antibodies were applied for detection, and nuclei were counterstained with ProLong® Gold Antifade mountant with DAPI (Thermo Fisher Scientific).

***In vivo* xenograft study**
Female athymic nude mice (5 weeks old; KOATECH, Republic of Korea) were maintained under specific pathogen-free conditions. HepG2 cells stably expressing mock vector, GREM2, RPL23, or GREM2 + RPL23 were injected subcutaneously into the flanks under isoflurane anesthesia. Body weights and tumor volumes were monitored twice per week. At the conclusion of the study, mice were sacrificed by CO₂ inhalation, and tumors were collected for further analysis.

**Statistical analysis**

All data are expressed as mean ± SD from at least three independent experiments. Group differences were assessed using two tailed *t*-test or one-/two-way ANOVA, with *p* < 0.05 considered significant.

**
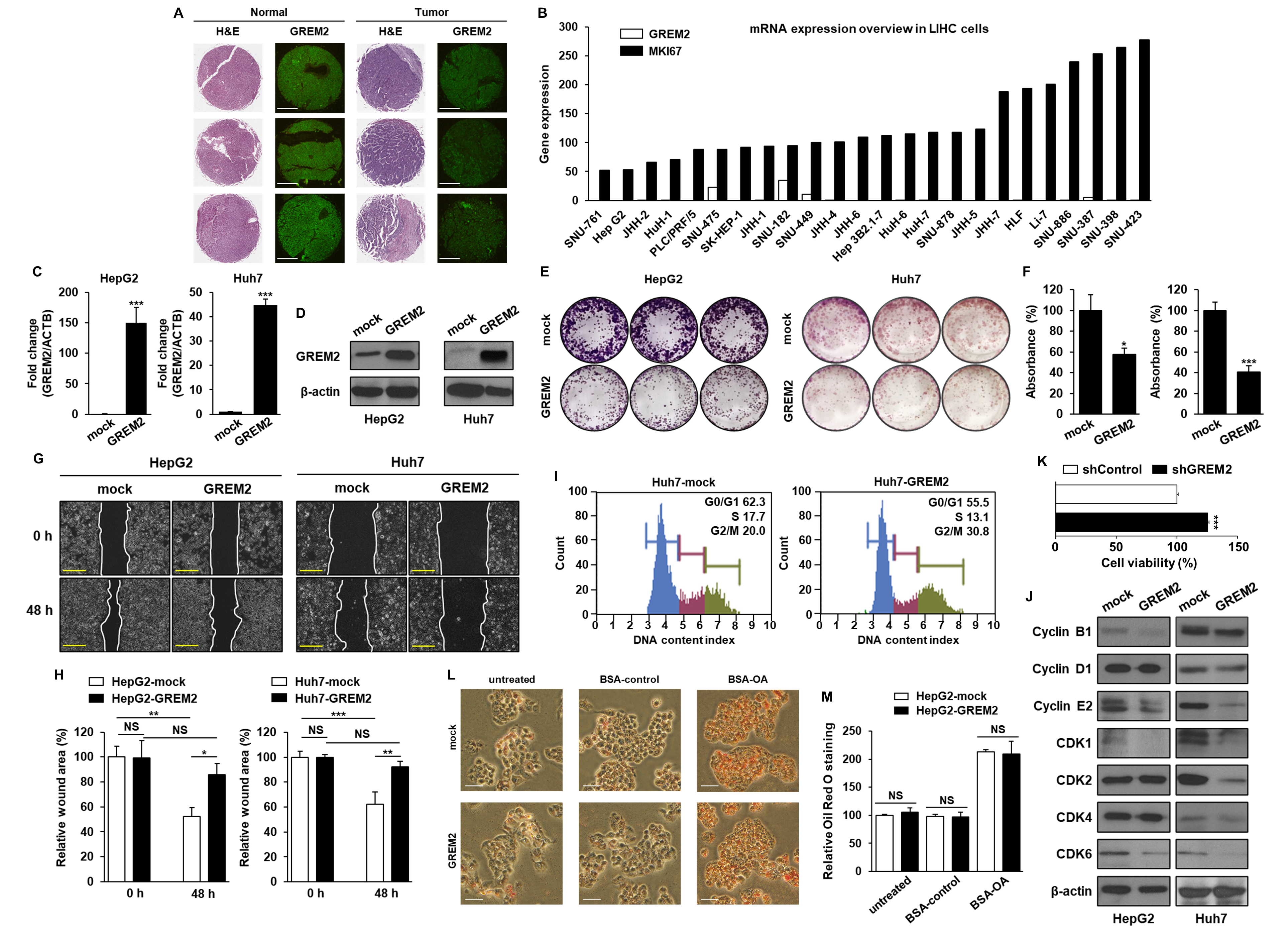
Figure S1** GREM2 inhibits cell proliferation in HCC. **(A)** Representative immunofluorescence images illustrate GREM2 staining in normal and tumor tissues; HCC microarray and H&E sections were obtained from TissueArray (Cat# LV481). **(B)** mRNA levels of GREM2 and MKI67 in HCC cell lines were assessed using the Expression Atlas database. **(C)** GREM2 overexpression in HepG2 and Huh7 cells was verified by qPCR. **(D)** Immunoblotting confirmed GREM2 protein levels. **(E, F)** Colony formation was quantified after 7–10 days in 6-well plates and visualized with crystal-violet staining. **(G, H)** Scratch-wound migration was evaluated in 12-well plates; scale bar = 500 µm. **(I)** Cell-cycle distribution was analyzed with a Muse™ Cell Cycle Kit. **(J)** Immunoblot analysis of cell cycle–related proteins was performed on each cell line. **(K)** Cell viability was determined using an MTT assay 24 h after seeding Huh7 cells transfected with either shControl or shGREM2 into 96-well plates. **(L, M)** HepG2 cells overexpressing either mock or GREM2 were treated with oleic acid (300 μM) for 72 h, followed by Oil Red O staining. Scale bar = 50 μm. **C, F**, **K,** two-tailed *t*-test; **H,** **M**, two-way ANOVA. * *p* < 0.05; ** *p* < 0.01; *** *p* < 0.001; NS, not significant.


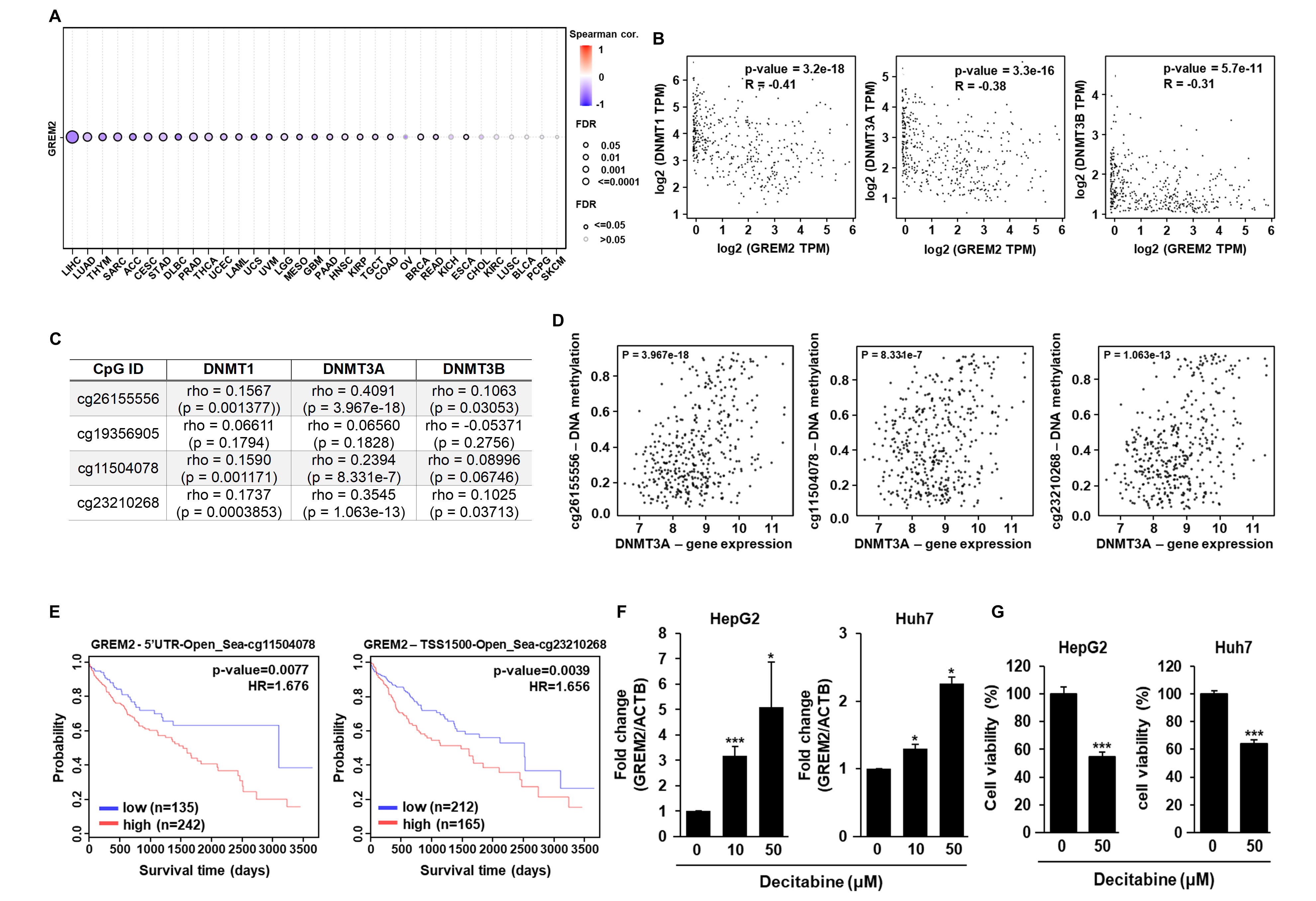


**Figure S2** Promoter hypermethylation causes reduced GREM2 expression in HCC. **(A)** GSCA pan-cancer analysis revealed the relationship between GREM2 promoter methylation and mRNA levels. **(B)** Correlations of GREM2 with DNA methyltransferases were examined using GEPIA2 (TCGA-LIHC & TCGA-normal). **(C)** Table showing the correlation between CpG methylation in the GREM2 promoter and the expression of DNMT family genes. **(D)** The correlation between DNMT3A expression and methylation levels of three CpG sites in the GREM2 promoter was analyzed using the UCSC genome browser. **(E)** MethSurv Kaplan-Meier plots showed HCC patient survival according to methylation of individual GREM2 CpG sites. **(F)** GREM2 mRNA was quantified by qPCR after 48 h treatment with 10 or 50 µM decitabine. **(G)** Cell viability after 50 µM decitabine for 48 h was assessed using MTT assay. Two-tailed *t*-test. * *p* < 0.05; *** *p* < 0.001.


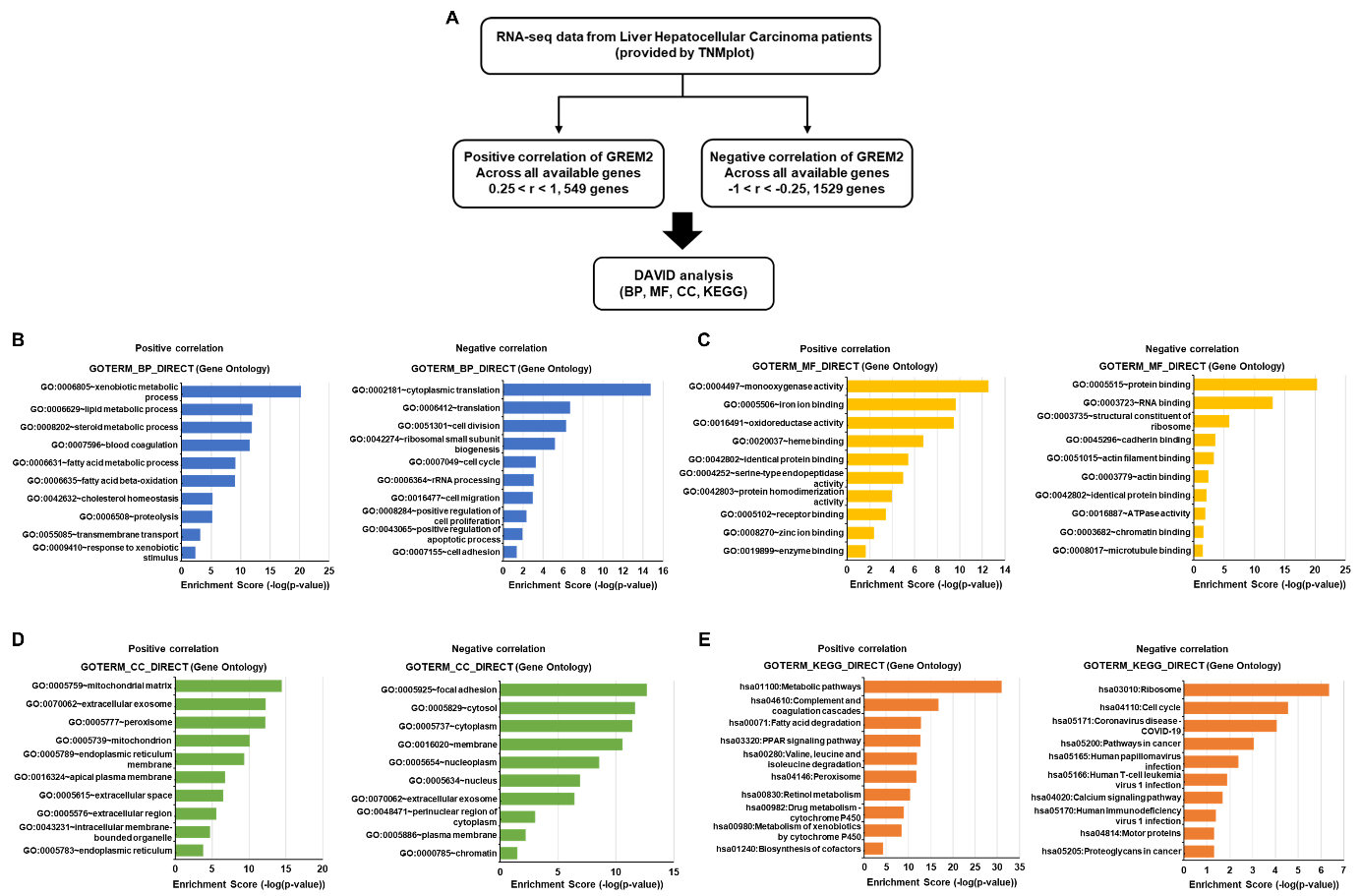


**Figure S3** GO pathways associated with GREM2 in HCC. **(A)** Workflow for selecting genes correlated with GREM2 from HCC RNA-seq data is shown. **(B-E)** DAVID analysis identified positively or negatively correlated genes in **(B)** Biological Process, **(C)** Molecular Function enrichment, **(D)** Cellular Component enrichment, and **(E)** KEGG pathway enrichment.

**
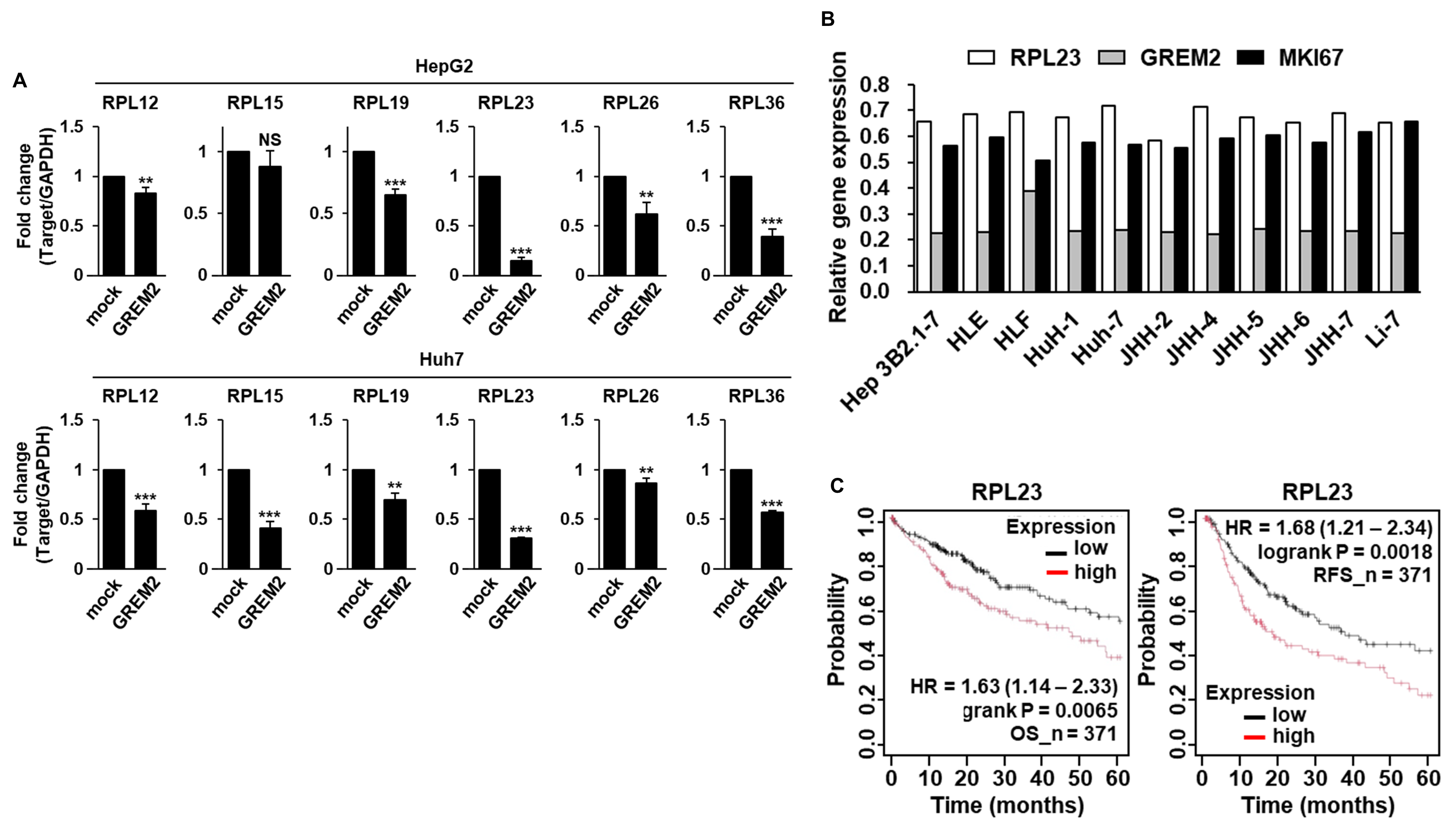
**

**Figure S4** GREM2 downregulates the expression of RPL23 in HCC cells. **(A)** Relative mRNA levels of representative ribosome-related genes were evaluated using qPCR. **(B)** GEMiCCL provided mRNA levels of RPL23, GREM2, and MKI67 across HCC cell lines. **(C)** Kaplan-Meier Plotter analysis showed overall and relapse-free survival according to RPL23 expression. Two-tailed *t*-test. ** *p* < 0.01; *** *p* < 0.001; NS, not significant.


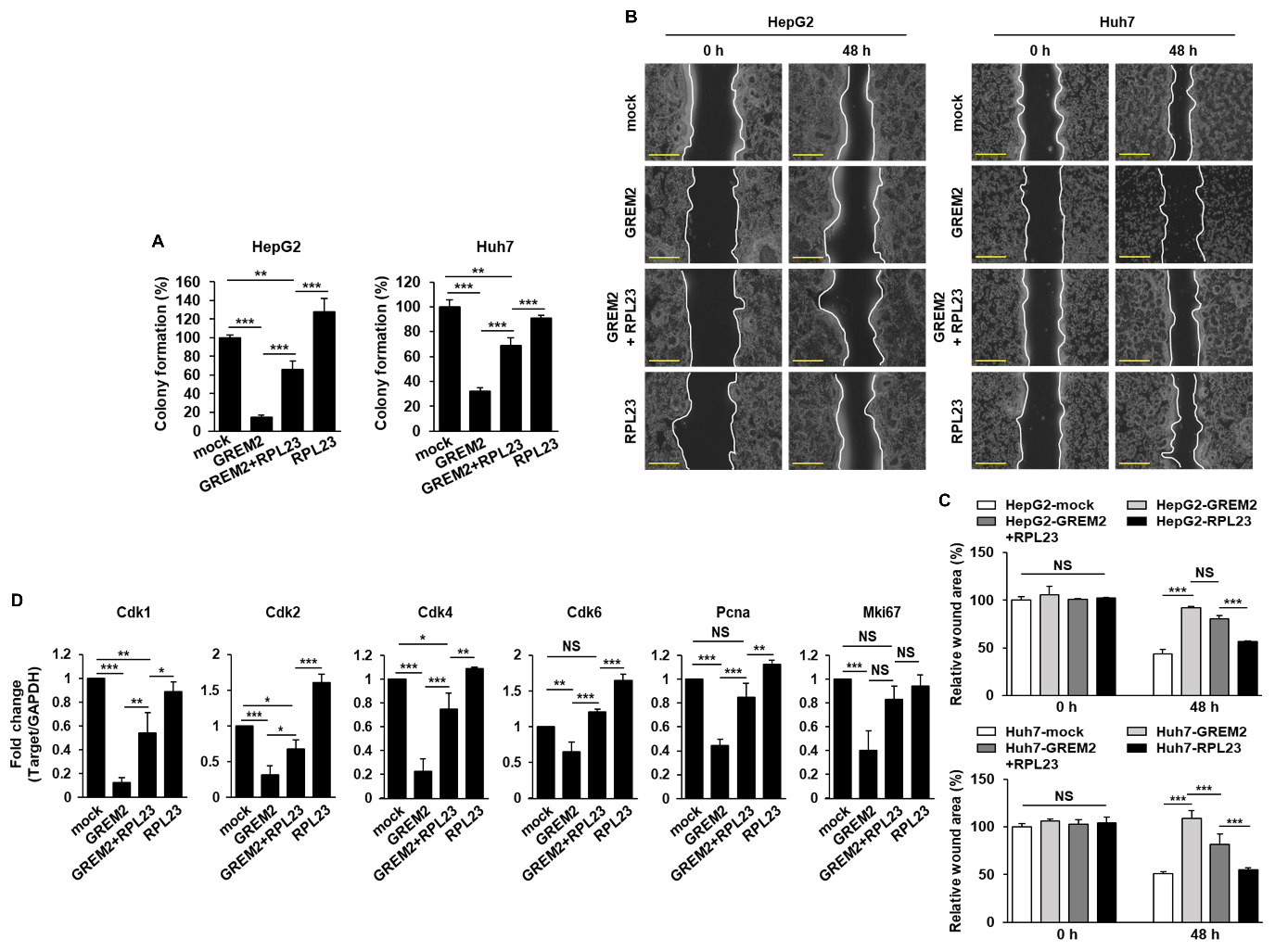


**Figure S5** Downregulation of RPL23 contributes to GREM2-mediated growth inhibition. **(A)** Crystal-violet colony-formation assay was performed after 7–10 days. **(B, C)** Scratch-wound migration was evaluated; scale bar = 500 µm. **(D)** qPCR quantified proliferation-associated genes in excised tumors. One-way ANOVA. * *p* < 0.05; ** *p* < 0.01; *** *p* < 0.001; NS, not significant.

**
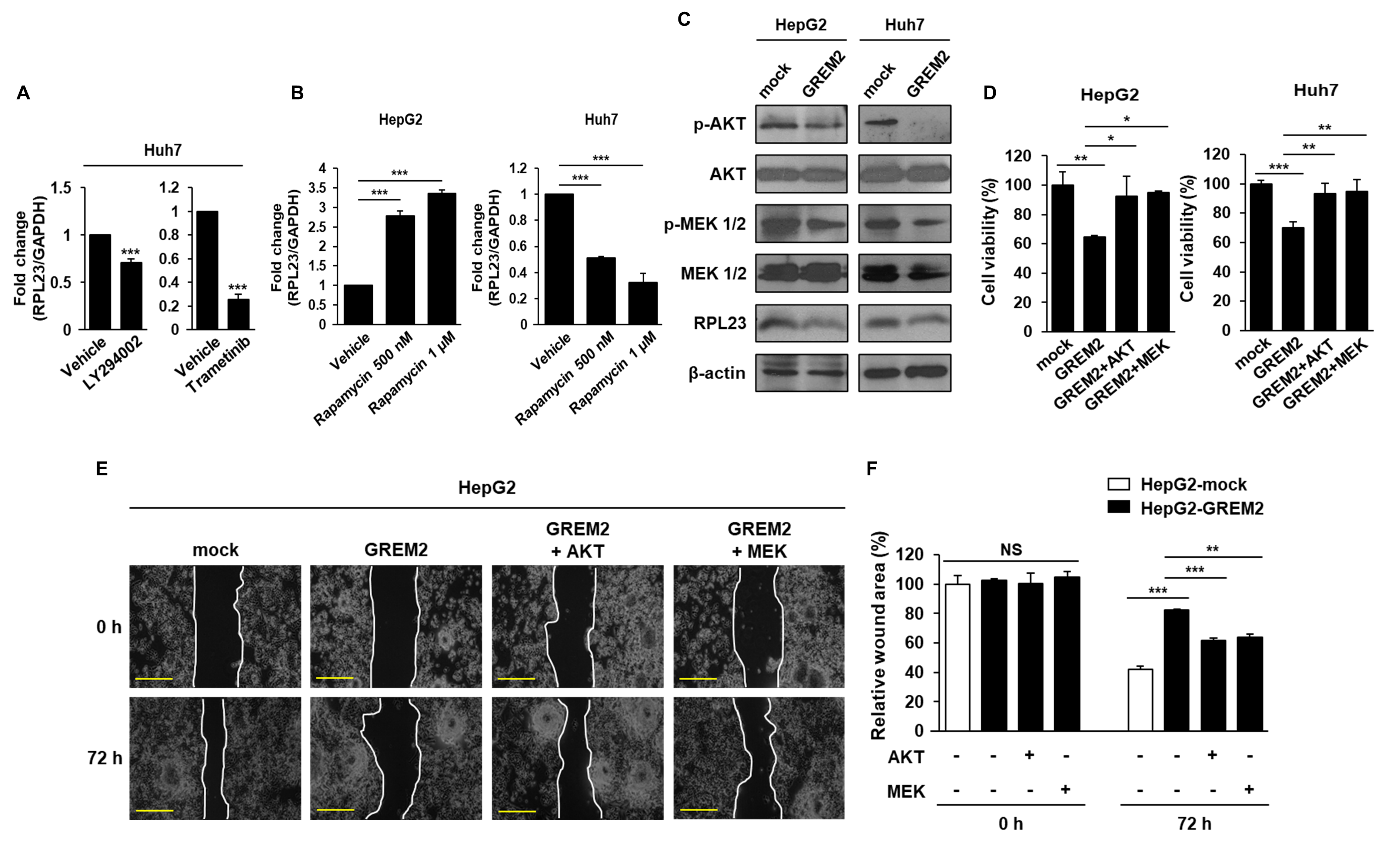
**

**Figure S6** GREM2 suppresses RPL23 through AKT/MEK signaling. **(A)** qPCR quantified target genes after 24 h treatment with LY294002 (25 µM) or trametinib (1 µM). **(B)** After treating each cell line with rapamycin (500 nM and 1 μM) for 48 h, mRNA expression of the corresponding genes was analyzed by qPCR. **(C)** Immunoblot analysis of phospho-AKT, phospho-MEK1/2, and RPL23 in stable lines was shown. **(D)** Cell viability was measured by MTT assay after 48 h. **(E, F)** Scratch-wound migration was analyzed; scale bar = 500 µm. **A**, **B,** two-tailed *t*-test; **D**, one-way ANOVA; **F**, two-way ANOVA. * *p* < 0.05; ** *p* < 0.01; *** *p* < 0.001; NS, not significant.
